# None of the Six SNPs of IL28B Could Predict Treatment Responses in Genotype 2 Chronic HCV Infected Patients by Propensity Score Matching Analysis

**DOI:** 10.1371/journal.pone.0048217

**Published:** 2012-11-16

**Authors:** Wen-Juei Jeng, Chun-Yen Lin, Ji-Yih Chen, Chang-Wen Huang, Chien-Hao Huang, I-Shyan Sheen

**Affiliations:** 1 Division of Hepatology, Department of Gastroenterology and Hepatology, Linkou Medical Center, Chang Gung Memorial Hospital, Taoyuan, Taiwan; 2 College of Medicine, Chang Gung University, Taoyuan, Taiwan; 3 Department of Rheumatology, Allergy and Immunology, Linkou Medical Center, Chang Gung Memorial Hospital, Taoyuan, Taiwan; 4 Institute of Clinical Medicine, National Yang-Ming University, Taipei, Taiwan; University of Sydney, Australia

## Abstract

**Background & Aims:**

A combination of pegylated interferon-alpha and ribavirin (PR) is the standard therapy for patients with chronic hepatitis C. The impact of polymorphism of interleukin-28B *(IL28B)* on sustained virological response (SVR) to PR has been well documented in patients with CHC genotype-1 (GT1), but it is controversial in genotype-2 (GT2) CHC patients. This study investigated the predictability of six single nucleotide polymorphisms (SNP) of *IL28B* on the treatment responses of PR in patients with CHC GT2.

**Method:**

197 CHC GT2 consecutive patients who received PR treatment in our prospective cohort were enrolled. Hepatitis C virus (HCV) genotyping, quantification of HCV-RNA and genotyping of the ten SNPs of *IL28B* were performed. Six SNPs of *IL28B* were chosen for analysis. The propensity score matching (PSM) analysis was applied using patients with CHC GT1 in another prospective cohort as a positive comparison to avoid covariate bias.

**Results:**

The distribution of the six SNPs was similar in GT1 and GT2 patients. Five of these SNPs had strong association with treatment responses in GT1 but not in GT2 patients. After PSM analysis, these five SNPs still showed strong association with rapid virological response (RVR), cEVR and SVR in GT1 and had no influence in GT2 patients. Furthermore, rs12979860 and baseline viral load were the predictors for both RVR and SVR in GT1 patients. However, only baseline viral load could predict RVR and SVR in GT2 patients. In addition, in patients without RVR, rs12979860 was the only predictor for SVR in GT1 but no predictor for SVR was found in GT2.

**Conclusions:**

The genetic polymorphisms of *IL28B* had no impact on treatment responses in GT2 patients.

## Introduction

Chronic hepatitis C (CHC) is a worldwide health issue affecting around 180 million people. The persistent inflammation caused by this virus may result in cirrhosis and hepatocellular carcinoma. A combination therapy of pegylated interferon-α (PegIFN) and ribavirin (PR) is a standard of care for chronic hepatitis C virus infection [1]. Current treatment strategy for CHC is to tailor the treatment guided by genotypes and on treatment viral response according to the roadmap concept [1], [2]. With these recommended strategies, a sustained virological response (SVR) rate could reach around 76–84% among patients with HCV genotype 2/3 (GT2/3) chronic infection, significantly better than the 42–52% in patients with HCV genotype 1 (GT1) [2].

Host genetic factors on the treatment efficacy for CHC have been proposed for a long time due to the ethnic differences in the treatment outcome. Better SVR rates, with 61–80% for GT1 and 80–95% for GT2/3 in Asian patients, were the main supporting evidence [3]–[12]. Recent genome-wide associated studies demonstrated strong evidence that single nucleotide polymorphisms (SNPs) of *Interluekin*-*28B (IL28B)* correlated significantly with SVR in GT1 patients when treated with PR [13]–[17]. Notably, the frequency of advantageous genotype of *IL28B* is highest in Asians and lowest in African-Americans, and it parallels the SVR rate in each population [13]. Furthermore, these genotypes of *IL28B* are also correlated with the spontaneous clearance of hepatitis C virus [14] and with viral kinetics during treatment in GT1 patients [17]. However, the results of the influences of these genetic variances on the treatment responses in GT2 patients were conflicting [18]–[22].

The aim of the present study was to use the propensity score matching (PSM) analysis to extend our understanding of genetic association in a cohort of patients with HCV GT2 chronic infection.

## Materials and Methods

### Patients

We conducted a retrospective analysis of our prospective cohort of consecutive adult Taiwanese patients with CHC GT1 and GT2 who visited the HCV team of the Department of Gastroenterology and Hepatology, Linkou Medical Center, Chang Gung Memorial Hospital. A universal 24-week regimen of PR (pegylated interferon alfa-α 2a 180 mcg/week or pegylated interferon alfa-α 2b 1.5 mcg/Kg/week, ribavirin 1000 mg-1200 mg/day) was reimbursed by the Bureau of National Health Insurance of Taiwan between February 2002 and December 2008. A total of 388 patients who provided written informed consent for this study, including genomic analysis, were enrolled. Patients with decompensated liver disease, hepatoma, co-infection with hepatitis B virus or with human immunodeficiency virus, or with apparent autoimmune hepatitis and alcoholic liver disease, were excluded from this cohort. Twenty-six percent of our patients had a history of blood transfusion, tattooing on skin or eyebrows, patterning of eyes, or piercing of earlobes. The remaining patients had no clear infection routes. Further, the distribution of infection routes in the GT1 and GT2 groups was similar.

**Table 1:** Baseline characteristics of chronic hepatitis C patients with genotype 1 and 2.

Variable	Total (N = 388)	GT 1 (N = 191)	GT 2 (N = 197)	P value
Gender, n (%)				0.128
male	235 (60.6)	123 (64.4)	112 (56.9)	
female	153 (39.4)	68 (35.6)	85 (43.1)	
Viral load, n (%)				0.044
<0.4×10^6^ IU/ml	205 (52.8)	91(47.6)	114 (57.9)	
≥0.4×10^6^ IU/ml	183 (47.2)	100 (52.4)	83 (42.1)	
Fibrosis stage, n (%)				0.410
F0-2	201 (51.8)	103 (53.9)	98 (49.7)	
F3-4	187 (48.2)	88 (46.1)	99 (50.3)	
Age, *y*	51.2±10.6	51.4±11.1	51.1±10.0	0.838
BMI, *Kg/m^2^*	24.5±3.3	24.5±3.3	24.6±3.2	0.603
HbA1c, *%*	5.7±1.1	5.6±1.0	5.7±1.1	0.430
ALB, *g/dL*	4.5±0.4	4.5±0.4	4.5±0.5	0.894
AST, *IU/L*	95±54	92±52	97±57	0.407
ALT, *IU/L*	153±106	149±107	157±106	0.256
GGT, *IU/L*	47±48	43±48	51±47	0.188
ALP, *IU/L*	82±44	80±25	85±57	0.668
Bilirubin, *mg/dL*	0.9±0.4	0.9±0.3	0.9±0.5	0.330
RVR, n (%)	293 (75.5)	133 (69.6)	160 (81.2)	0.008
cEVR, n (%)	316 (81.4)	147 (77.0)	169 (85.8)	0.025
SVR, n (%)	311 (80.2)	131 (68.6)	180 (91.4)	<0.001

Continuous variables were expressed as mean ± standard deviation; BMI: body mass index; HbA1c: glycohemoglobin; ALB: albumin; AST: aspartate transaminase; ALT: alanine transaminase; GGT: gamma-glutamyl transpeptidase; ALP: alkaline phosphatase; RVR: rapid virologic response; cEVR: complete early virologic response; SVR: sustained virologic response.

All patients received pre-treatment liver biopsies that were evaluated by one liver-specialized pathologist using the Metavir scoring system. HCV genotypes were determined by a genotype-specific probe-based assay in the 5′ untranslated region (LiPA; Innogenetics, Ghent, Belgium).

For analysis of the responses to treatment, subjects with SVR were defined as undetectable serum HCV-RNA 24 weeks after cessation of treatment. The subjects with rapid virological response (RVR) were defined as undetectable serum HCV-RNA 4 weeks after starting treatment. Complete early virological response (cEVR) was defined as undetectable serum HCV-RNA at week 12 after treatment.

The HCV antibody was tested by the Architect anti-HCV (Abbott Diagnostics, Irving, TX, USA). The HCV-RNA was quantitated by the VERSANT HCV RNA 3.0 Assay (bDNA) (Bayer Diagnostics, Tarrytown, NY, USA; lower limit of detection at 3200 copies/ml) or COBAS TaqMan HCV Test (TaqMan HCV; Roche Molecular Systems Inc., Branchburg, NJ, USA; lower limit of detection at concentrations of 15 IU/ml). Those with undetectable RNA by bDNA were reassayed by Cobas Amplicor HCV v2.0 kit (Roche Molecular Systems, Pleasanton, CA, USA; lower limit of detection at concentrations of 100 copies/ml).

**Figure 1:**
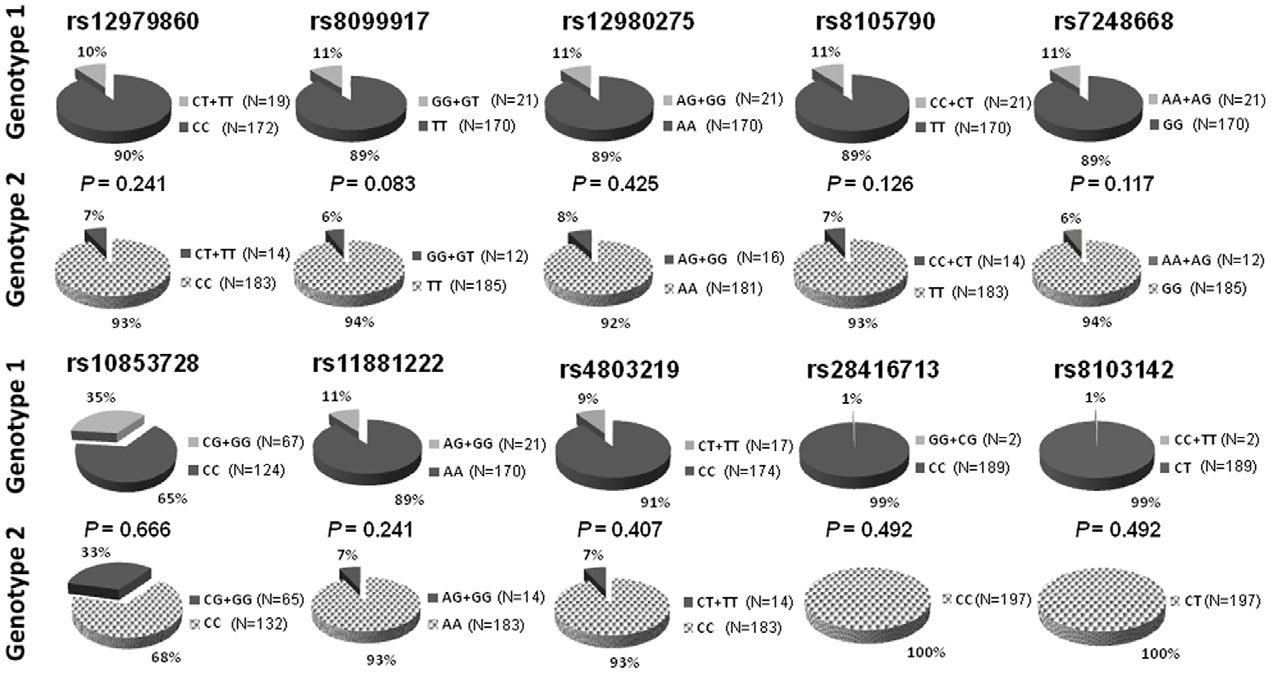
The distribution of the ten SNPs of IL 28B between GT1 and GT2. The genotypic distributions of the 10 SNPs of IL28B were similar in GT1 and GT2 patients (p>0.05).

**Figure 2:**
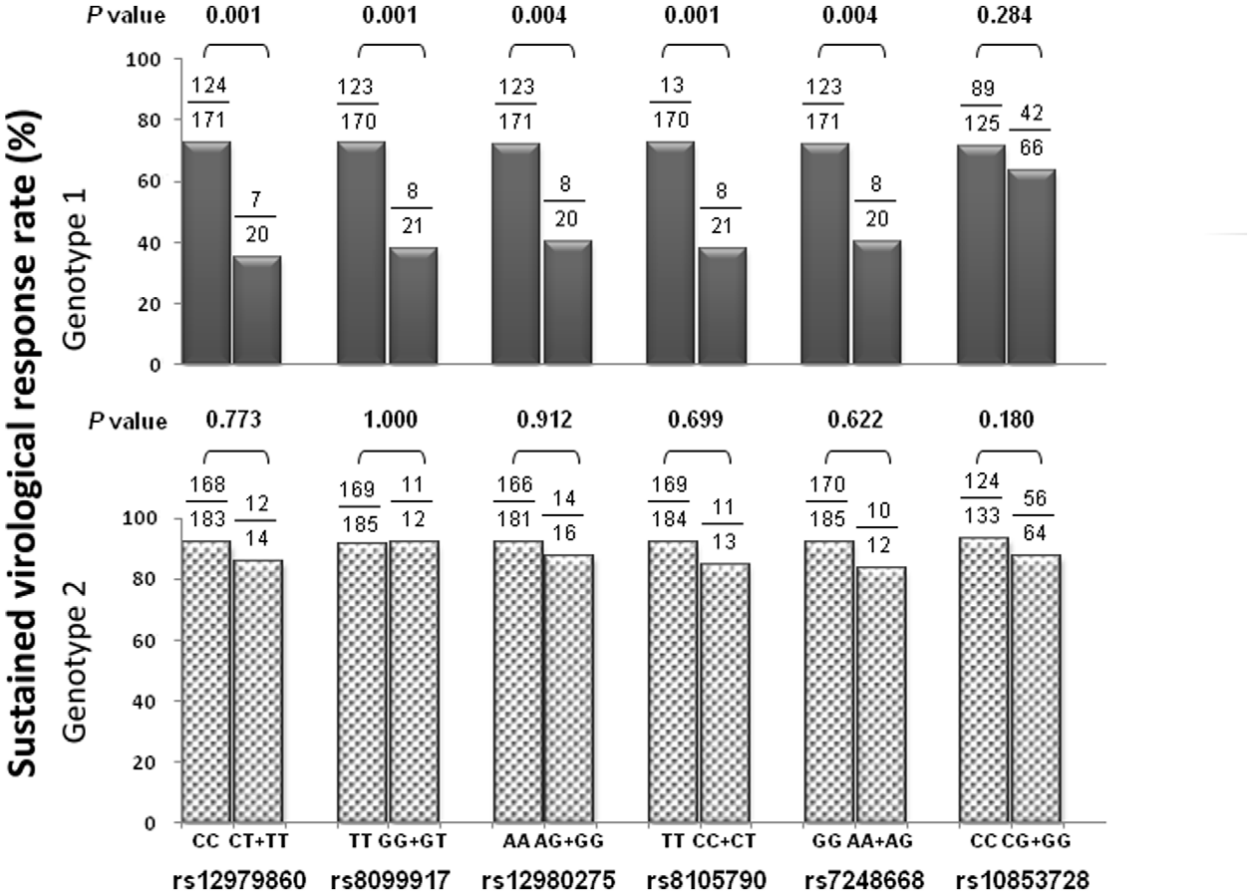
The correlation of SNPs with SVR in GT1 and GT2 patients. Six SNPs were chosen for analysis (rs12979860, rs8099917, rs12980275, rs8105790, rs7248668, 10853728). Five (rs12979860, rs8099917, rs12980275, rs8105790, and rs7248668) of these six SNPs revealed significant association with SVR in GT1 patients while none of them revealed any relationship between these SNPs and the SVR in GT2 patients.

**Table 2:** Baseline characteristics of chronic hepatitis C patients with genotype 1 and 2 after PSM.

Variable	Total (N = 290)	GT 1 (N = 145)	GT 2 (N = 145)	P value
Male, n (%)	180 (62.1)	90 (62.1)	90 (62.1)	1.000
RNA≥0.4×10^6^ IU/ml, n (%)	134 (46.2)	67 (46.2)	67 (46.2)	1.000
Fibrosis stage 3–4, n (%)	118 (40.7)	59 (40.7)	59 (40.7)	1.000
Age, *y*	50.3±10.7	50.0±11.4	50.7±10.1	0.597
BMI, *Kg/m^2^*	24.4±3.0	24.5±3.2	24.4±2.9	0.678
HbA1c, *%*	5.7±1.2	5.7±1.1	5.7±1.2	0.699
ALB, *g/dL*	4.5±0.4	4.5±0.4	4.5±0.4	0.278
AST, *IU/L*	93±55	92±54	94±56	0.976
ALT, *IU/L*	156±116	153±117	159±116	0.557
GGT, *IU/L*	46±50	44±54	47±46	0.802
ALP, *IU/L*	79±25	78±25	79±26	0.574
Bilirubin, *mg/dL*	1.0±0.4	1.0±0.3	0.9±0.5	0.164
RVR, n (%)	208 (71.7)	89 (61.4)	119 (82.1)	<0.001
cEVR, n (%)	228 (78.6)	102 (70.3)	126 (86.9)	0.001
SVR, n (%)	229 (79.0)	99 (68.3)	130 (89.7)	<0.001

### Genomic DNA Extraction and *IL28 B* Genotyping

#### Nucleic acid isolation

Anti-coagulated peripheral blood was obtained from patients with CHC GT1. Genomic DNA was isolated from EDTA anti-coagulated peripheral blood using the Pure gene DNA isolation kit (Gentra Systems, Minneapolis, MN) as previously described [23].

**Figure 3:**
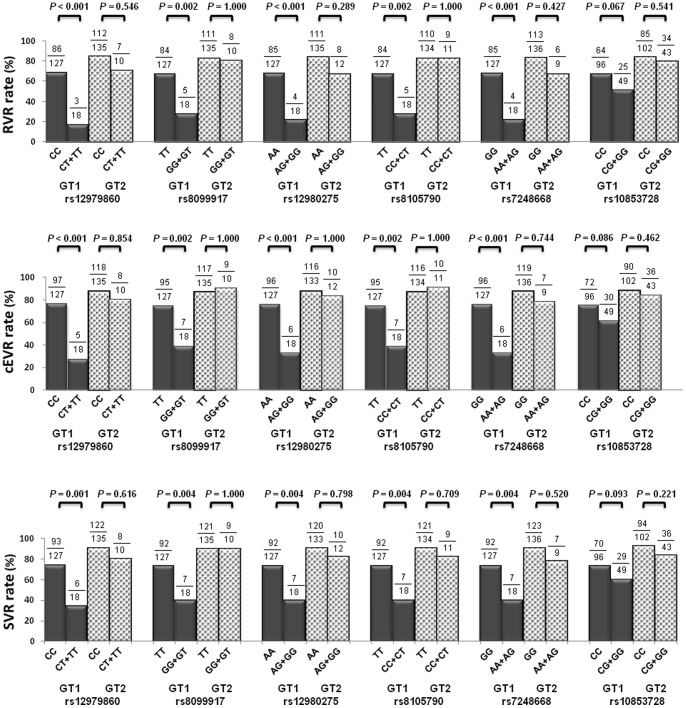
The role of SNPs in RVR (3A), EVR (3B) and SVR (3C) between GT1 and GT2 after PSM analysis. Five out of these SNPs (rs12979860, rs8099917, rs12980275, rs8105790, rs7248668) showed significant association with RVR, cEVR and SVR in GT1 patients but none of these SNPs had relationship with treatment responses in GT2 patients.

**Table 3:** Predictors for RVR, cEVR and SVR in both GT1 and GT2 patients after PSM.

	Variables	UV			MV			
		OR	P value	OR		95% CI	P value	
RVR	GT1							
	rs12979860	10.49	<0.001	13.0		3.3–51.0	<0.001	
	rs8099917	5.08	0.004					
	rs12980275	7.08	0.001					
	rs8105790	5.08	0.004					
	rs7248668	7.08	0.001					
	rs10853728	1.92	0.069					
	HbA1c	0.81	0.161					
	Viral load	3.83	<0.001	4.5		2.1–9.7	<0.001	
	GT2							
	HbA1c	0.99	0.995					
	Viral load	2.61	0.034	2.6		1.1–6.3	0.034	
cEVR	GT1							
	rs12979860	8.41	<0.001					
	rs8099917	4.67	0.003					
	rs12980275	6.19	0.001					
	rs8105790	4.67	0.003					
	rs7248668	6.19	0.001					
	rs10853728	1.90	0.088					
	BMI	0.89	0.039					
	GGT	0.99	0.068					
	ALP	0.99	0.058					
	RVR	264.00	<0.001	264.0		33.6–2075.0	**<**0.001	
	Viral load	4.05	<0.001					
	GT2							
	BMI	0.82	0.032					
	GGT	1.01	0.214					
	ALP	0.98	0.068					
	RVR	265.50	<0.001	265.5		31.3–2250.3	<0.001	
	Viral load	1.72	0.277					
SVR	GT1							
	rs12979860	5.47	0.002	4.4		1.2–16.8	0.028	
	rs8099917	4.13	0.007					
	rs12980275	4.13	0.007					
	rs8105790	4.13	0.007					
	rs7248668	4.13	0.007					
	rs10853728	1.86	0.095					
	Age	0.97	0.044	0.96		0.9–0.99	0.033	
	BMI	0.86	0.010					
	Gender	2.09	0.043					
	Viral load	4.85	<0.001	4.2		1.7–10.3	0.002	
	Fibrosis stage	2.28	0.024					
	RVR	7.14	<0.001	3.8		1.6–9.1	0.003	
	cEVR	5.36	<0.001					
	GPT	1.00	0.127					
	ALB	2.13	0.140					
	GT2							
	Age	0.99	0.822					
	BMI	0.78	0.015	0.8		0.7–1.0	0.049	
	Gender	5.38	0.006	0.2		0.1–0.7	0.009	
	Viral load	9.15	0.005	7.4		1.5–35.7	0.013	
	Fibrosis stage	4.70	0.011					
	RVR	2.60	0.110					
	cEVR	2.79	0.112					
	GPT	1.01	0.030					
	ALB	4.08	0.044					

UV: Univariate analysis; MV: Multivariate analysis.

**Table 4:** Predictors for SVR in both GT1 and GT2 patients with or without RVR after PSM.

Variables	With RVR					Without RVR				
	UV		MV			UV		MV		
	OR	P value	OR	95% CI	P value	OR	P value	OR	95% CI	P value
GT-1										
rs12979860	1.02	0.989				4.20	0.045	4.2	1.0–17.1	0.045
rs8099917	1.37	0.788				3.18	0.111			
rs12980275	1.55	0.708				3.67	0.071			
rs8105790	1.37	0.788				3.18	0.111			
rs7248668	1.85	0.607				2.27	0.219			
rs10853728	1.03	0.965				2.00	0.215			
Age	0.98	0.359				0.96	0.094			
Gender	3.83	0.028	0.3	0.1–1.0	0.054	1.56	0.431			
BMI	0.883	0.137				0.85	0.109			
Viral Load	4.63	0.013	4.2	1.2–14.3	0.024	2.54	0.107			
Fibrosis stage	2.83	0.080				1.59	0.397			
GPT	1.01	0.089				1.00	0.939			
GGT	1.02	0.312				0.99	0.544			
GT-2								No significant variable		
Age	1.01	0.838				0.95	0.350			
Gender	3.04	0.100				1.48	0.220			
BMI	0.77	0.048				0.86	0.369			
Viral Load	6.38	0.023	8.8	1.5–50.8	0.015	9.00	0.065			
Fibrosis stage	3.58	0.075	5.3	1.1–26.7	0.043	12.80	0.038			
GPT	1.01	0.039	1.0	1.0–1.1	0.010	1.01	0.466			
GGT	1.01	0.540				1.00	0.902			

#### Taqman-based assay for the genotyping of SNPs

The oligonucleotide sequence flanking ten *IL28B* polymorphisms were designed as primer for Taqman allelic discrimination assays. Both allele specific primers were labeled with a fluorescent dye (FAM and VIC) and used in the PCR reaction. Aliquots of the PCR product were genotyping with allele specific probe of SNPs using real time PCR (ABI) [24]. IL28B variants rs12979860 C>T, rs8099917 T>G, rs12980275 A>G, rs8105790 T>C, rs7248668 G>A, rs10853728 C>G were diagnosed from whole blood samples using validated pyrosequencing™ assays. We used these six SNPs from ten of our previous report [23] because of significant deviations from the Hardy-Weinberg equilibrium in two SNPs and used one as the representative of the haplotype (three SNPs).

### Ethics Statements

All patients in this study had provided written informed consent. This study protocol conformed to the ethical guidelines of the 1975 Declaration of Helsinki and was approved by the ethical committees of Chang Gung Memorial Hospital.

### Statistical Analysis

Chi-square test or Fisher’s exact probability test was used to compare the categorical variables where appropriate. Continuous variables were compared using Mann-Whitney U test or Student’s *t*-test. Univariate and multivariate logistic regression analyses for the significant independent predictors of virological responses were conducted using patients’ demographics, clinical variables and *IL28B* SNPs. Variables that achieved statistical *P* value<0.10 on univariate analysis were entered into multivariate logistic regression analysis. The propensity score matching (PSM) analysis [24]–[26] was applied for bias reduction in the investigation of the association between SNPs and the treatment outcomes in GT1 and GT2 patients. Variables possibly influencing the treatment outcomes, including gender, baseline HCV-RNA viral load, fibrosis stage, glycohemoglobin (HbA1c) and basal metabolic index (BMI) were used to generate a propensity score ranging from 0 to 1 by logistic regression. Nearest available matching on the estimated propensity score was used to select the GT1 and find the GT2 subject with the closest propensity score. One hundred and forty-five well-matched pairs of GT1 and GT2 patients were obtained. All *P* values less than 0.05 by the two-tailed test were considered statistically significant. All statistical analyses were performed with the SPSS statistical software for Windows (version 16, SPSS. Inc., Chicago, IL, USA).

## Results

### None of the SNPs of *IL28B* were Associated with SVR in GT2 Patients, though they were in GT1 Patients

The demographic characteristics of these patients are shown in Table 1. There were 191 GT1 and 197 GT2 patients. More patients of GT1 had high viral load (HCV-RNA≥0.4×10^6^ IU/ml) than GT2 patients (GT1 vs. GT2∶52.4% vs. 42.1%, *p* = 0.044). Other demographic data were comparable between these two groups. Similar to previous literature, the rates of RVR, cEVR and SVR were significantly lower in GT1 patients than GT2 patients (Table 1). The genotypic distributions of the six SNPs of IL28B were similar in GT1 and GT2 patients (Figure 1). Five (rs12979860, rs8099917, rs12980275, rs8105790, and rs7248668) of these six SNPs revealed significant association with SVR in GT1 patients. These SNPs had no influence on the SVR in GT2 patients (Figure 2).

After balancing the covariates in GT1 and GT2 patients to reduce the bias possibility by the PSM (Table 2), all the treatment responses of GT1 were significantly lower than GT2 (SVR: 68.3% vs. 89.7%, *P*<0.001; RVR: 61.4% vs. 82.1%, *P*<0.001; cEVR: 70.3% vs. 86.9%, *P* = 0.001). As for the relationship between the SNPs of *IL28B* and the treatment responses (Figure 3), five out of the six SNPs showed significant association with RVR, cEVR and SVR in GT1 patients, but none of these SNPs had any relationship with treatment responses in GT2 patients.

### Different Predictors for RVR, cEVR and SVR in GT1 and GT2 Patients

Because the SNPs of *IL28B* had different impacts on the treatment responses in GT1 and GT2 patients, we therefore analyzed the possible predictors for treatment responses in these two PSM adjusted patient groups. In the prediction of RVR, rs12979860 and baseline HCV RNA viral load were the independent predictors in GT1 patients, while baseline HCV-RNA viral load was the only predictor in GT2. In the cEVR prediction analysis, only RVR was the predictor in both GT1 and GT2 patients. As for the SVR, rs12979860, age, baseline viral load and RVR were the predictors in GT1 patients, while BMI, gender and baseline viral load were the predictors in GT2 patients (Table 3).

In our previous report [27] **,** we found that in GT1 patients with RVR, only baseline viral load could predict the SVR. As for GT1 patients without RVR, rs12979860 became the predictor for SVR. Similar observation was found in GT1 patients in this PSM analysis. However, in GT2 patients with RVR, baseline RNA, fibrosis and ALT levels were the predictors for SVR. Interestingly, none of these factors including SNPs of *IL28B* were associated with SVR in GT2 patients without RVR (Table 4).

## Discussion

The treatment of CHC has evolved rapidly. Response to tailored treatment planning has been emphasized after a roadmap concept is established [2]. The discovery of the host genome factors of *IL-28B* was a milestone since it demonstrated and explained the influence of genetic variations and ethnicity on the efficacy of interferon-based therapy for patients with GT1 CHC [28], [29]. In our previous report, by genotyping six SNPs of *IL28B*, we had shown a significant correlation of genetic polymorphisms of *IL28B* with SVR in GT1 Asian patients [27]. The present study revealed that none of the six SNPs had association with either RVR or SVR in Asian GT2 patients, even by PSM approach.

Though the impact of polymorphisms of *IL28B* on the treatment responses of GT1 patients is very clear, the impact of this genetic polymorphism on the treatment responses in GT2 patients is still controversial [18]–[22]. Rauch et al. had reported that genotype of *rs8099917* had no significant impact on the SVR in Swiss patients infected with HCV GT2 or 3 [15]. Yu et al. [19] found no influence of *rs8099917* on SVR of Asian HCV GT2 patients, but a higher RVR rate in those with *TT rs8099917*. On the other hand, in Italian patients, Mangia [18], Lindh [20] and Sarrazin [21] reported that the genotype of *rs12979860* had significant association with SVR in patients with HCV GT2 or GT3 infection. Kawaoka [22] found that the initial viral load and *rs8099917* genotype are significantly independent predictors of SVR in genotype 2 patients in Japan.

The three reports [18], [20], [21] about the impact of IL28B on the treatment outcomes of chronic hepatitis C GT2 or GT3 patients showed IL28B polymorphism was associated with SVR in the 24-week treatment and the effect was only observed in the non-RVR patients. In one of them, Sarrazin et al. [21] claimed only GT3 but not GT2 patients had a significantly higher SVR rate in the patients with advantageous allele. The other two did not further divide their patients into GT2 and GT3 groups [18], [20]. However, genotype 2 and 3 are not really the same [30], [31]. Here, our patients are all GT2 HCV infections, and no associations between treatment outcomes (SVR or RVR) and rs12979860 polymorphism were found. In our study, the polymorphism of IL28B SNPs influences the RVR in GT1 patients rather than GT2 patients.

In addition, the distribution of the homozygous responding allele was around 40% in Caucasians but it is the dominant pattern (about 90%) in our patients. Furthermore, the dosage of pegylated interferon and ribavirin varied among Western studies (PEG-INTRON: 1.0 or 1.5 mcg/kg/week; ribavirin: 600–1400 mg/day), and treatment duration varied from 12 weeks to 24 weeks. In the present study, we used a fixed dosage of PegIFN and RBV (PEG-INTRON: 1.5 mcg/kg/week or pegasys 180 mcg/week; ribavirin: 1000–1200 mg/day), and a fixed duration (24 weeks). These factors may also contribute to the different conclusions in different studies.

To investigate the association between SNPs and treatment outcome in an observational database rather than a randomized control trial, a propensity score matching analysis was an important method used to reduce patient selection bias [24]–[26] and to avoid imbalance covariates between groups. Through PSM, we used GT1 patients as a comparison group to match covariates that had been reported to influence treatment responses between GT1 and GT2 patient groups. The results still demonstrated the SNPs (rs12979860, rs8099917, rs12980275, rs8105790, and rs7248668) had no relationship with SVR in GT2 patients, though they had significant impact on SVR in GT1 patients. Therefore, it is fair to claim that the genotype of *IL28B* had great impact on the treatment response only in GT1 patients but not GT2 patients. In addition, till now only two SNPs of *IL28B*, rs8809917 and rs12979860, had been investigated in GT2 patients. We studied six SNPs of *IL28B* that had been published before [13]–[16] and found that none of these SNPs had any relationship with the treatment responses in GT2 patients.

In previous studies [7], [32], [33] and ours focusing on GT1 patients [27], the genetic variants of IL28B SNP had a more significant impact on SVR in patients without RVR rather than those with RVR. However, in the present studies, the genetic variants of IL28B SNP did not influence the treatment outcome in both the RVR group and non-RVR group in GT2 patients using propensity score matching analysis. Similar results were found by Yu [19], also done in an Asian population, that the SNP of *IL28B* had no impact on SVR in GT2 patients, whether RVR had been achieved or not.

Much better response rates (RVR, cEVR and SVR) have been demonstrated in GT2 patients than GT1 patients. The stronger sensitivity of HCV GT2 to PR might offset the increasing response to PR by *IL28B*, even for those GT2 patients with no RVR, though two reports from Europe [18], [20] showed that the SNP of *IL28B* had a significant impact on SVR in GT2 or GT3 patients who did not achieve RVR. The majority of their patients were genotype 3 and the response to PR treatment was not as good in genotype 3 as it was in genotype 2. Therefore, further studies on the association of IL28B and SVR in only genotype 2 Caucasian patients are needed.

The limitation of this study is that the sample size was not big enough. Further external validation in larger populations as well as in Western countries is necessary. In addition, hidden bias may remain even after PSM because of matching only the controls for observed variables (to the extent that they are perfectly measured).

In conclusion, our data demonstrated that none of the six SNPs of *IL28B* had any relationship with SVR in GT2 patients, irrespective of the overall number of patients, or in those with or without RVR.
